# RESTORE: A Prospective Multinational Registry of Patients with Genetically Confirmed Spinal Muscular Atrophy - Rationale and Study Design

**DOI:** 10.3233/JND-190451

**Published:** 2020-03-20

**Authors:** Richard S. Finkel, John W. Day, Darryl C. De Vivo, Janbernd Kirschner, Eugenio Mercuri, Francesco Muntoni, Perry B. Shieh, Eduardo Tizzano, Isabelle Desguerre, Susana Quijano-Roy, Kayoko Saito, Marcus Droege, Omar Dabbous, Farid Khan, Lydie Renault, Frederick A. Anderson, Laurent Servais

**Affiliations:** a Department of Pediatrics, Division of Neurology, Nemours Children’s Hospital, Orlando, FL, United States; b Department of Neurology, Stanford University Medical Center, Stanford, CA, United States; c Departments of Neurology and Pediatrics, Columbia University Irving Medical Center, New York, NY, United States; d Clinic for Neuropediatrics and Muscle Disease, University Medical Center Freiburg, Freiburg, Germany; e Department of Paediatric Neurology and Nemo Clinical Centre, Catholic University, Rome, Italy; f Department of Developmental Neuroscience, University College London, London, UK; g Department of Neurology, David Geffen School of Medicine at UCLA, Los Angeles, CA, United States; h Department of Clinical and Molecular Genetics, Hospital Valle Hebron, Barcelona, Spain; i Hôpital Necker Enfants Malades, APHP, Paris; j Garches Neuromuscular Reference Center (GNMH), APHP Raymond Poincare University Hospital (UVSQ), Garches, France; k Institute of Medical Genetics, Tokyo Women’s Medical University, Tokyo, Japan; l AveXis, Inc., Bannockburn, IL, United States; m AveXis, Switzerland GmbH, Zurich, Switzerland; n Center for Outcomes Research, University of Massachusetts Medical School, Worcester, MA, United States; o Oxford Neuromuscular Center, Oxford University, UK

**Keywords:** Spinal muscular atrophy, multinational, prospective, registry, outcomes, rare disease, long-term follow-up, NCT04174157

## Abstract

**Background:**

Dramatic improvements in spinal muscular atrophy (SMA) treatment have changed the prognosis for patients with this disease, leading to important new questions. Gathering representative, real-world data about the long-term efficacy and safety of emerging SMA interventions is essential to document their impact on patients and caregivers.

**Objectives:**

This registry will assess outcomes in patients with genetically confirmed SMA and provide information on the effectiveness and long-term safety of approved and emerging treatments.

**Design and Methods:**

RESTORE is a prospective, multicenter, multinational observational registry. Patients will be managed according to usual clinical practice. Both newly recruitedSMAtreatment centers and sites involved in existing SMA registries, including iSMAC, Treat-NMD, French SMA Assistance Publique- Hôpitaux de Paris (AP-HP), Cure-SMA, SMArtCARE, will be eligible to participate; *de novo*; sites already participating in another registry may be included via consortium agreements. Data from patients enrolled in partnering registries will be shared with the RESTORE Registry and data for newly diagnosed patients will be added upon enrollment. Patients will be enrolled over a 5-year period and followed for 15 years or until death. Assessments will include SMA history and treatment, pulmonary, nutritional, and motor milestones, healthcare resource utilization, work productivity, activity impairment, adverse events, quality of life, caregiver burden, and survival.

**Status:**

Recruitment started in September 2018. As of January 3, 2020, 64 patients were enrolled at 25 participating sites.

**Conclusions:**

The RESTORE Registry has begun recruiting recently diagnosed patients with genetically confirmed SMA, enabling assessment of both short- and long-term patient outcomes.

## INTRODUCTION

Spinal muscular atrophy (SMA) is a neurogenetic disorder caused by loss of or pathogenic variants in the survival motor neuron 1 gene (*SMN1*) on chromosome 5q13, which leads to reduced SMN protein levels and a selective dysfunction of motor neurons. SMA is an autosomal recessive spectrum of disorders, with an estimated incidence of 1:10,000 live births, 60 percent of which is the most severe form, SMA Type 1. SMA is the leading cause of infant mortality among genetic diseases [1]. *SMN2* is a paralogous gene that is present in all patients and is considered a phenotype modifier. Multiple copies of *SMN2* are associated with milder phenotypes [2]. Over the last 10 years, there have been numerous publications describing the natural history of patients with SMA and the effects of available supportive therapies [3– 14].

Until recently, supportive medical care was the mainstay of treatment for patients with SMA. Results from recent clinical trials of gene therapy, antisense oligonucleotides, and small molecules have demonstrated that these interventions can alter the course of disease [15– 20]. At the same time, standard of care guidelines have been published that support proactive intervention [15, 21, 22].

There is a need for long-term efficacy and safety information from patients receiving both existing and new therapies to document the evolving trajectory of SMA and the impact of these treatments on patient and caregiver outcomes as well as on the healthcare system. These findings promise to enhance data from long-term extensions of completed and ongoing clinical trials [23, 24]. In addition, evolving therapies raise several questions with direct impact on daily practice, such as the best prognostic factors, early identification of likely responders, reasonable expectations regarding treatment outcomes, durability of therapeutic response, and approaches to combining established and emerging therapies. Results from clinical trial extensions can be supplemented and extended by longitudinal registry data, which can play an important role in redefining the natural history of a disease resulting from the availability of disease-modifying therapies [25].

The RESTORE Registry will assess outcomes of patients with a diagnosis of 5q SMA with biallelic deletion of *SMN1*, with the primary objective of assessing contemporary SMA treatments, including effectiveness, short- and long-term safety, and overall patient survival. RESTORE will also assess pharmacoeconomic and quality-of-care outcomes of contemporary and evolving SMA treatments, including healthcare resource utilization and caregiver burden. It was designed to overcome the recognized limitations of single-product registries [25] including difficulty in combining results for research purposes due to lack of comparability in data collected and limited access to data by academic researchers.

## MATERIALS AND METHODS

### Design

RESTORE is a prospective, multicenter, multinational, non-interventional observational study governed by an international steering committee of SMA experts who are committed to ensuring the quality of these data and to sharing findings through publication and presentation of Registry data. It is the first global SMA registry, consolidating data from multiple countries, established since the advent of gene therapy for SMA. All patients will be managed at participating clinical sites according to best available practices. Clinical care will not be dictated by a research protocol. No additional visits or investigations will be performed beyond those consistent with normal clinical practice. Patients will be enrolled over a 5-year period and followed for 15 years, or until death.

Together with individual SMA treatment centers recruited *de novo,* participating centers may include those involved in existing and evolving SMA registries, including the International SMA Consortium (iSMAC) [24, 26], Treat-NMD [27– 29], the French SMA registry, Cure SMA [30] and SMArtCARE [31]. Data from patients already enrolled in partnering registries will be transferred to the Registry database, assuming ethical approval and patient consent are obtained as required. Where inclusion of individual patient data is not feasible, aggregate data may be shared. Data for newly diagnosed patients will be added as they are enrolled by their physicians (Table 1). Motor phenotype data will be collected by trained physical therapists. Avoidance of potential duplication of counting patients among these different registries will be accomplished by assigning each patient a Global Unique Identifier. The RESTORE Registry is sponsored by AveXis (a Novartis company), manufacturer of onasemnogene abeparvovec, a gene therapy for SMA. The registry data are owned by the sponsor, which has developed bylaws providing for control of publications under the authority of a Steering Committee comprised of academic physicians and scientists with expertise in SMA.

**Table 1:** Data sources used for the RESTORE Registry

•Individual *de novo* clinical sites
•Existing SMA Consortia
•Managed Access Programs
•Expanded Access Programs
•Post Marketing Surveillance (required follow up)

### Ethical considerations

RESTORE is being conducted in accordance with established research principles, local treatment practices and regulations, and guidelines of the International Council on Harmonisation. At centers recruited *de novo*, physicians will be asked to provide documentation and approvals per local regulations. Once a site is activated, physicians will consent patients using an Informed Consent Form (ICF) that has been approved by their Independent Ethics Committee/Institutional Review Board in accordance with local practices and regulations. Prior to any data collection, a written ICF and a privacy statement will be signed by the parent/guardian; and, where appropriate, by the patient. All information obtained during the conduct of the Registry with respect to the patient’s identity or state of health will be treated as confidential. Prior to any disclosure of protected personal information, a signed written agreement will be obtained from the patient or his/her legal representative.

### Patients

All enrolled patients must have 5q SMA diagnosed at <18 years of age that is genetically confirmed (bi-allelic loss or pathological variants of the *SMN1* gene) [32]. Subtypes of SMA will be categorized based on age at onset, number of SMN2 copies, and highest acquired motor function (e.g., independent sitting and ambulation). All patient recruitment and data management will be conducted in accordance with FDA/EMA requirements.

During a 5-year enrollment period, RESTORE will document the long-term safety and effectiveness of gene therapy, along with that of evolving and emerging SMA treatments, including up to 15-years of follow-up. To minimize selection bias, consecutive eligible patients will be enrolled from participating clinical sites. To obtain adequate patient numbers to support this new observational study, an international collaboration of existing SMA registries and *de novo* clinical sites is being created. Patients will be recruited worldwide to provide sufficient data to document differences between patients who receive a variety of treatments.as they evolve during the 15-year duration of this registry.

### Registry procedures

Patient care will follow usual SMA treatment practices in each country and participating clinical site. No additional diagnostic or monitoring procedures will be required beyond the routine clinical practices at each participating clinic or hospital.

### Treatment

The choice of ongoing medical treatment for the duration of the Registry will be made independently by the physician in the course of regular patient care and will not be influenced by participation in this Registry. Physicians are free to add or withdraw any medication but will continue to monitor each patient for the full 15 years, until death, or until the patient is withdrawn from the Registry, which may occur at the discretion of the patient or the patient’s parent/legal representative or physician. No treatments will be provided by the sponsor as part of participation in this Registry.

### Data acquisition and variables assessed

No mandatory visits, tests, or assessments are required for participation in this Registry. All follow-up visits will be scheduled and conducted according to each participating site’s usual clinical practices.

#### Data acquisition

Sources that will generate data for this Registry include consortia, individual clinical sites, managed- and expanded-access Programs (MAP/EAP), and post-marketing study obligations. Additional prospective data for SMA patients that meet RESTORE eligibility criteria may be extracted from existing registries that agree to share their information (Table 1).

Additional data will be collected from *de novo* study sites by local study coordinators who will complete online electronic data capture based on information in the patient’s medical chart. Additionally, for *de novo* patients, self-reported data will be collected using standardized patient reported outcome questionnaires and caregiver surveys. Study variables (e.g., socio-demographic characteristics, history of SMA, pulmonary assessments, ventilatory support, nutritional assessment, motor milestone assessments) and standardized patient and caregiver reported outcomes assessed in the registry are listed in Table 2.

**Table 2:** Study variables collected in the RESTORE Registry

Category	Variables
Confirmation of Eligibility,	•Date of informed consent for study enrollment
Socio-demographics, Study Status	•Eligibility Assessment
	•Socio-demographic characteristics:
	∘ Year of Birth
	∘ Gestational age
	∘ Gender
	∘ Race
	∘ Ethnicity (US)
	•Withdrawal of consent:
	∘ Date of withdrawal
	∘ Reason for withdrawal
Clinical Characteristics of Patient	•Medical history:
	∘ History of SMA:
	■ Date and age of diagnosis
	■ Genetic status
	■ *SMN2* copy number
	■ Point mutation
	■ Weight at diagnosis of SMA
	■ Length/height at diagnosis of SMA
	∘ Other medical history
	∘ Family history:
	■ Maternal and paternal genetic test results
Treatments	■ Onasemnogene abeparvovec treatment (if applicable):
	∘ Date of treatment, dose
	•Prednisolone treatment (onasemnogene abeparvovec group only)
	•Nusinersen treatment:
	∘ Dose and frequency
	∘ Start and stop dates
	•Other concomitant medications:
	∘ Dose and frequency
	∘ Start and stop dates
Patient Assessments	•Pulmonary assessments:
	∘ Was it performed, normal/abnormal, if abnormal details
	•Ventilatory support:
	∘ Cough assist details
	∘ Non-invasive details
	∘ Invasive details
	•Nutritional assessment:
	∘ Use of non-oral procedure to administer food:
	∘ Details including start/stop, volume, frequency, caloric intake
	•Motor milestone assessments:
	∘ Developmental milestones
	∘ Children’s Hospital of Philadelphia Infant Test of Neuromuscular Disorders (CHOP INTEND)
	∘ Hammersmith Infant Neurological Examination (HINE)
	∘ Hammersmith Functional Motor Scale
	•Laboratories
	∘ Liver function test
Hospitalization and Healthcare	•Emergency room visits and hospitalizations:
Resource Utilization	∘ Date of hospitalizations
	∘ Reason of hospitalizations
	•Other therapies and visits
	•Insurance type (in US)
Patient/Caregiver Reported	•Work Productivity and Activity Impairment Questionnaire SMAv2
Outcomes	•Zarit Burden Interview
	•PedsQL Child report
	•PedsQL Parent report concerning child
Serious Adverse Events and Death	•Serious adverse events (and adverse events of special interest):
	∘ Start and stop dates
	•Death:
	∘ Date of death
	∘ Primary cause of death

#### Data analysis

The analysis population will consist of all patients enrolled. The primary analysis will be a summary of outcomes stratified according to the therapy a patient is receiving at the time of enrollment. Descriptive statistics will be presented for the primary analysis. Consistent with the observational nature of this registry, no formal *a priori* hypothesis testing will be performed. Continuous variables will be summarized using the number of observations, mean, standard deviation, median, minimum, and maximum. Categorical data will be summarized using counts and percentages. Incidence rates (per 10,000 person-years) and 95% confidence intervals for adverse events of special interest will be calculated. Survival will be evaluated using Kaplan-Meier methods. All rates and confidence intervals for individual responses will be performed on actual data. Missing observations will not be imputed in the analysis of individual questions or items.

## DISCUSSION

RESTORE is a prospective, treatment-independent, collaborative global registry. It will recruit patients from a variety of settings and backgrounds allowing assessment of short- and long-term outcomes representative of the contemporary treatment of patients with a confirmed diagnosis of SMA. To permit evaluation of patients who receive gene therapy with onasemnogene abeparvovec, this Registry will recruit patients who were previously treated in a formal clinical study or EAP/MAP.

The primary objective of the Registry is to gather long-term follow-up information on patients’ outcomes that cannot be collected in the time frame of a typical clinical trial. Contemporary follow-ups from EAP programs [31, 33, 34] or clinical trials [15, 17, 18] do not exceed 2 years and include only patients treated with a single approach. The potential importance of inclusion of patients receiving multiple therapies is underscored by an example from the treatment of Duchenne Muscular Dystrophy, in which a registry enrolling a patient who received several therapies could permit comparison of the long-term efficacies of deflazacort and prednisone [35], and better assess the efficacy of daily treatment when compared to alternative regimens [36].

In addition, long-term data collection from a large patient sample may provide important insights regarding prognostic factors, characteristics of best responders to therapies, and estimation of the duration of unsuccessful treatment after which a patient can be considered a non-responder. Another very important question that can only be assessed by long-term follow-up is the need for and the cost-effectiveness of treating pre-symptomatic patients. Even if there are few or no questions about this approach in patients with 2 or 3 *SMN2* copies [37], this question will undoubtedly arise for patients with 4 copies, reflecting the creation of numerous newborn screening programs [38– 40]. The issue of treating presymptomatic patients has also been raised in late-onset Pompe disease [41] and this question should be addressed generally. Another important consideration related to treatment of asymptomatic patients, who are potential candidates for gene therapy, is the persistence of transgene expression, which can be determined over long-term follow-up of Registry patients.

Review of the design of existing registries suggests that the variables evaluated in RESTORE will complement and extend analyses of existing registries, including provision of important information about the clinical course of SMA in patients receiving new treatments. Ongoing discussions with the leaders of existing SMA registries indicate that there is a large overlap in the types of data collected and a willingness, consistent with the charter of each registry, to share data for the benefit of patients, families, regulatory agencies, and clinical researchers.

Given that RESTORE will provide data describing the clinical management of patients with SMA across multiple countries, there is a potential limitation due to variations in the standard of care across countries or regions, and variation in treatments based on cultural norms with the potential for missing data for some measures. This has been noted as a potential issue for all rare disease registries [25]. This is particularly important for the most severely affected patients, since they constitute the group for which the extent of medical care is the most important. In patients with less severe disease, such as those who are ambulatory, differences in standards of care are less likely to directly and significantly impact patient outcomes. Another limitation of this Registry is the absence of standard training for neuromuscular therapists, and more generally, the lack of standardization across SMA medical assessment.

RESTORE was designed to overcome the recognized limitations of single-product registries [25, 42] including difficulty in combining results for research purposes due to lack of comparability in data collected and limited access to data by academic researchers. When patients are exposed to multiple treatments, particularly at different clinics, partial data for individual patients may be stored in multiple registries. Consequently, their full experience across treatments cannot be appreciated by studying data in a single-product registry.

Comparisons of patient data may be limited due to large differences in population characteristics. Addressing this challenge in RESTORE may present an opportunity to answer critical questions about the importance of variations in patients and systems of care.

Because patients will not be randomized, statistics will be limited to comparisons of means, frequencies, and temporal trends. The prognostic factors for key milestones, including sitting for the SMA type 1 patients, or walking for type 2 and type 3 patients, will be investigated by comparing proportions of sitters and walkers according to multiple parameters, including disease duration, number of *SMN2* copies, baseline conditions, etc. Our focus will be on assuring the representativeness of patients, completeness of the information collected for each patient, and the validity of study data.

Due to uncontrolled variations in scheduling follow-up care, data cannot be collected at fixed intervals. Even if all families are offered similar standards of care, some may refuse all or a portion of this support, and variations in Registry findings will reflect the heterogeneity of care delivered. In addition, some patients may choose to enroll in controlled clinical trials during the follow-up period. Loss to follow up, independent of patient status, represents another challenge for data collection and assessment.

Strengths of our design include a focus on enrolling consecutive eligible patients who were genetically diagnosed, representing a broad range of evolving SMA care practices and outcomes across many countries and health care systems. This should provide a robust characterization of the course of disease for patients with SMA receiving the full range of contemporary and evolving treatments and supportive care. Additional strengths of an observational study design include the option to modify the registry design as standards of care are advanced and experience is gained from the care of patients receiving interventions that greatly extend the lifespans of SMA patients. During the planned 15-year duration of the Registry, additional variables may be added to the data collection forms, leading to important insights into questions that were not anticipated in the original study design.

In conclusion, RESTORE is a disease registry aimed at assessing the safety and effectiveness of new and evolving treatments for SMA during a 5-year enrollment period and followed for up to 15 years. This project is being undertaken with an open and collaborative approach, with the aim of combining multinational and multi-institutional resources. In addition to providing information about the long-term efficacy and safety of emerging SMA treatments that promise to revolutionize the course of disease for patients with SMA, RESTORE will also assess pharmacoeconomics (e.g., healthcare resource utilization, caregiver burden) and quality-of-care associated with contemporary and evolving SMA treatments. Thus, RESTORE will provide valuable insights into both the effectiveness and value of SMA therapies, representing an essential resource to inform treatment decisions and improve patient outcomes.
